# Enzymatically Hydrolyzed Poultry By-Product Supplementation, Instead of Fishmeal, Alone Improves the Quality of Largemouth Bass (*Micropterus salmoides*) Back Muscle without Compromising Growth

**DOI:** 10.3390/foods12183485

**Published:** 2023-09-19

**Authors:** Changguo Yi, Dongyu Huang, Heng Yu, Jiaze Gu, Hualiang Liang, Mingchun Ren

**Affiliations:** 1 Wuxi Fisheries College, Nanjing Agriculture University, Wuxi 214081, China13815427718@163.com (J.G.); lianghualiang@ffrc.cn (H.L.); 2 Key Laboratory of Integrated Rice-Fish Farming Ecology, Ministry of Agriculture and Rural Affairs, Freshwater Fisheries Research Center, Chinese Academy of Fishery Sciences, Wuxi 214081, China

**Keywords:** largemouth bass, protein source replacement, muscle quality, enzymatically hydrolyzed poultry by-products

## Abstract

This study was designed to investigate the effects of enzymatically hydrolyzed poultry by-products (EHPB) on the growth and muscle quality of largemouth bass. Different concentrations of EHPB (0.00, 3.10, 6.20, 9.30, and 12.40%) were added to replace fishmeal (0.00 (control), 8.89 (EHPB1), 17.78 (EHPB2), 26.67 (EHPB3), and 35.56% (EHPB4)), respectively, in dietary supplementation. The results revealed that the growth performance and muscle amino acid and fatty acid remained unaltered in EHPB1 (*p* > 0.05). EHPB1 showed significant reduction in muscle hardness, gumminess, chewiness, and muscle fiber count and exhibited a significant increase in muscle fiber volume. The decrease in muscle hardness, gumminess, and chewiness means that the muscle can have a more tender texture. The expression of protein metabolism-related genes reached the highest levels in EHPB1 and EHPB2 (*p* < 0.05). The mRNA levels of *s6k* and *igf-1* in EHPB2 and EHPB1 were significantly lower than those in the control group. Compared to the control group, the expression of muscle production-associated genes *paxbp-1* was higher in EHPB1, and *myod-1*, *myf-5*, and *syndecan-4* were higher in EHPB2. The mRNA levels of muscle atrophy-related genes, in EHPB4 and EHPB2, were significantly lower than those in the control group. Therefore, the EHPB1 group plays a role in promoting the expression of genes related to muscle formation. In summary, replacing 8.89% of fishmeal with EHPB in feed has no effect on growth and may improve back muscle quality in largemouth bass.

## 1. Introduction

Global population growth and improved living standards have driven increasing demand for aquatic products. Consequently, the demand for high-quality aquatic products is growing [1]. The fishing of natural resources alone is insufficient to meet the human demand for aquatic products, necessitating expansion of the scale of aquaculture to ensure a sustainable supply of high-quality protein sources for humans [2]. However, a high dependence on the feed industry is a significant constraint on the development of aquaculture [3]. Large-scale aquaculture requires the consumption of large amounts of compound feed to meet the demand for aquaculture. Increased feed complexity necessitates more feed ingredients, especially fishmeal [4]. The global scarcity of fishmeal has prompted the exploration of alternative land-based sources of raw materials. Researchers have made progress in the research and development of new aquatic feed [5]. Additional studies are required to investigate the effects of replacing land-based raw materials in fish feed on factors such as palatability, growth, muscle quality, and immunity in fish.

Several studies have explored alternatives to fishmeal in aquatic animal feeds [6,7,8]. Simultaneously, several issues have been brought to light. Several fishmeal replacements, especially plant-based raw materials, contain anti-nutritional factors that adversely affect digestion, absorption, and utilization in animals [9,10]. Therefore, carnivorous fish are commonly fed animal proteins, single-cell proteins, or processed plant-based proteins as alternatives to fishmeal. In largemouth bass (*Micropterus salmoides*), animal proteins exhibit better apparent digestibility than plant proteins [11]. Enzymatic hydrolysis of poultry by-products (EHPB) is the process of breaking down poultry by-products using enzymes. A complex enzyme, mainly papain, was added to the poultry by-products composed of poultry racks, feathers, and blood. EHPB was obtained by enzymatic hydrolysis at a certain temperature. Poultry by-products share a composition similar to fishmeal. Enzymolysis enhances the absorption and utilization of small peptides and other substances that are decomposed from poultry by-products [12]. Enzymatic hydrolysis partially converts insoluble proteins in poultry by-products into a water-soluble form, which facilitates absorption in the intestine [13]. Previous studies have confirmed that EHPB could improve the digestibility, immunity, and growth performance and antioxidant capacity of turbot (*Scophthalmus maximus*) [14]. Therefore, EHPB serves as an ideal protein source for reducing the use of fishmeal in aquatic feeds.

Muscles of aquatic animals serve as the primary protein source in aquaculture. Muscle quality determines the success of aquatic products to some extent. The muscle quality of aquatic animals is influenced by various factors, including the growth stage [15], feed composition [16], culture pattern [17], and aquaculture environmental factors [18]. Biochemical processes during muscle-to-meat conversion significantly affect the quality of fresh muscle. These modifications affect various distinct characteristics of muscle, including appearance, nutritional composition, and texture. Therefore, modifying the muscle fiber characteristics of living animals may be a viable approach for regulating fresh muscle quality [19]. Recent studies have demonstrated that substitution of dietary protein sources can affect the muscle quality of various aquatic species, such as mirror carp (*Cyprinus carpio*) [20], tilapia (*Oreochromis niloticus*) [21], and largemouth bass (*Micropterus salmoides*) [22]. In addition, animal protein sources, particularly poultry by-products, can improve the muscle quality of aquatic animals, including protein and fatty acid content and chewability [23,24]. In the context of fishmeal replacement, there is still a lack of understanding of the influence of various new protein sources on the muscle quality of aquatic products. Currently, research on the effects of EHPB as a feed protein source on the muscle quality of aquatic animals is limited. Moreover, the effect of EHPB on the muscle quality of largemouth bass remains unclear. Therefore, there is a pressing need to investigate the effect of EHPB as a potential replacement for fishmeal in largemouth bass.

Largemouth bass (*Micropterus salmoides*) is a carnivorous fish native to Canada and the United States. The global cultivation of this species is prevalent because of its delicate meat and delicious taste. Decreased intermuscular counts facilitate feeding. Fishmeal is a crucial component of largemouth bass farming, accounting for a significant portion of the feed costs [25]. Several studies have investigated alternative feed options for largemouth bass [26,27,28]. Furthermore, there is a growing concern regarding the muscle quality of largemouth bass [29,30]. To date, no study has examined the use of EHPB as a replacement for fishmeal in largemouth bass diets, and the effect of EHPB on largemouth bass muscle quality remains unexplored. Therefore, this study aimed to investigate the potential of EHPB as a replacement for fishmeal in largemouth bass and its effect on the muscle quality of fish.

## 2. Materials and Methods

### 2.1. Experimental Management

The site and fish used in the experiment were provided by the Wuxi Fishery College of the Nanjing Agricultural University. The range of largemouth bass meal replacement was established based on previous research [31,32]. The feed formulae are listed in Table 1. Five isonitrogenous and isolipid diets (containing 0.00% (control), 3.10% (EHPB1), 6.20% (EHPB2), 9.30% (EHPB3), and 12.40% (EHPB4) EHPB, replacing 0.00%, 8.89%, 17.78%, 26.67%, and 35.56% fishmeal, respectively) were used to feed largemouth bass. EHPB comes from Tongwei Company, and the enzyme used in enzymatic hydrolysis is a complex enzyme based on papain. The ingredients were filtered through an 80-mesh screen, mixed, and granulated using an F-26 (II)-type granulator (South China University of Technology). The granules obtained were subsequently dried and stored at −20 °C for future use, as described in our previous study [22]. A sample of 150 largemouth bass, with an average weight of 240 ± 12 g, were randomly allocated into 15 cages measuring 2 m^3^ each (five experimental groups, three parallel groups). Feeding was stopped for 24 h before the commencement of the experiment. During the experiment, largemouth bass were fed twice daily in the morning and evening to reach apparent satiety. The amount of food consumed was recorded. During the experiment, the dissolved oxygen levels exceeded 6 mg/L, the pH ranges from 7.5 to 8.0, the ammonia nitrogen level was below 0.2 mg/L, and the temperature range was 30 ± 2 °C.

### 2.2. Sample Collection

**Figure 1:**
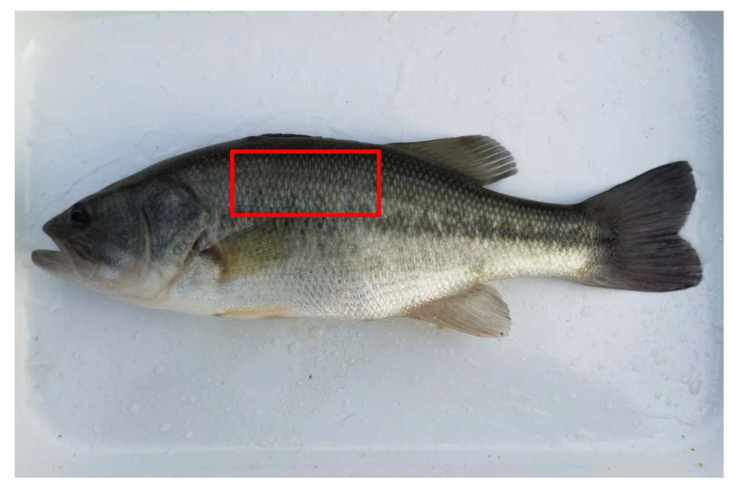
Sampling site of largemouth bass back muscles.

Samples were collected 68 days after the initiation of the culture experiment. A pre-sampling fasting period of one day facilitated the emptying process of the largemouth bass intestines. All caged largemouth bass were weighed. Largemouth bass were anesthetized using MS-222 before sampling. Subsequently, each fish was weighed individually. Nine of them were collected from each cage for blood and back muscle. Back muscle texture parameters were immediately identified. Sample sections were fixed using 5% paraformaldehyde. Plasma was collected after blood centrifugation. The remaining samples were cryopreserved using liquid nitrogen and subsequently stored at −80 °C. The sampling site for largemouth bass back muscle is shown in Figure 1.

### 2.3. Experimental Detection

#### 2.3.1. Feed and Muscle Analyses

Feed and muscle analyses were performed using the AOAC method [33]. The general steps included moisture testing in an oven at 105 °C. Crude protein content was determined using the Kjeldahl method. Crude lipid content was measured using a Soxhlet extractor. Crude ash content was determined using the muffle furnace method. Subsequently, the amino acid and fatty acid components of the back muscles were analyzed based on the findings of the texture analysis. Table 2 presents the instrumental details.

#### 2.3.2. Muscle Texture, Slice Analysis, and Muscle Component Analysis

Hardness, cohesiveness, gumminess, springiness, chewiness, and resilience of the back muscles were measured using a texture analyzer. The experimental group with the greatest difference from the control group was selected for slice comparison and muscle component analysis. The target area of the selected tissue was imaged at a magnification of 200× using a microscope. After imaging, Image-Pro Plus 6.0 analysis software was used for the analysis, with millimeter as the standard unit of measurement. The formulae used for the analysis and calculations are as follows:

Area of a single muscle fiber = total muscle fiber area/total number of muscle fibers

Muscle fiber density = total muscle fiber/total muscle fiber area

The method of muscle composition analysis was the same as that of feed.

Muscle amino acids were detected using an amino acid analyzer based on acid hydrolysis. Muscle fatty acids were analyzed using a gas chromatograph according to the standard GB: 5009.168-2016 [34]. Table 2 presents the instrumental details.

#### 2.3.3. Fluorescent Quantitative PCR

The total RNA was extracted and quantified using a spectrophotometer. The PCR instrument was run using a set program. The instruments and reagents used in this study were consistent with those described in our previous studies [35,36] and listed in Table 2. The primers used are listed in Table 3, with *β-actin* serving as the internal reference gene. The relative standard curve method was used to analyze the expression of related genes.

#### 2.3.4. Plasma Biochemistry

Plasma samples were analyzed using a blood biochemical analyzer. The detection indices included albumin (ALB), alanine aminotransferase (ALT), aspartate aminotransferase (AST), total cholesterol (TC), triglycerides (TG), glucose (GLU), total protein (TP), and alkaline phosphatase (ALP). The instruments and reagents used in our research (listed in Table 2) were similar to our previous studies [38,39].

### 2.4. Data Analysis

The experimental data were analyzed using SPSS 25.0, with one-way ANOVA and *t*-tests. The results are reported as the mean ± S.E.M. Duncan’s multiple comparison test was performed to determine significant differences, with a significance level of *p* < 0.05. Significant differences were denoted with different letters.

## 3. Results

### 3.1. Growth Performance

Table 4 presents the growth performance. The inclusion of dietary EHPB1 increased the final body weight (FBW) and specific growth rate (SGR) to some extent and reduced the feed coefficient rate (FCR). The inclusion of EHPB2 and EHPB3 in the diet significantly decreased FBW and SGR and increased FCR (*p* < 0.05). Furthermore, this effect was exacerbated by higher levels of EHPB supplementation. In addition, when the dietary EHPB content reached EHPB4, the survival rate (SR) was significantly lower than that in the control group (*p* < 0.05).

### 3.2. Plasma Biochemistry

Table 5 presents the plasma biochemical analysis conducted on largemouth bass. The ALB concentration was significantly improved in EHPB1 compared with the control group and peaked in the EHPB3 group (*p* < 0.05). The highest TG level was observed in the EHPB1 group, followed by a subsequent decline. The EHPB1 group exhibited significantly higher levels of ALT, AST, TC, and GLU than the control group (*p* < 0.05). TP levels were significantly elevated in the EHPB1 group and peaked in the EHPB2 group, which was significantly higher than the content in the control group (*p* < 0.05). The ALP level was lowest in EHPB2, which was significantly lower than that in the control group (*p* < 0.05).

### 3.3. Muscle Texture, Slice Analysis, and Muscle Component Analysis

**Figure 2:**
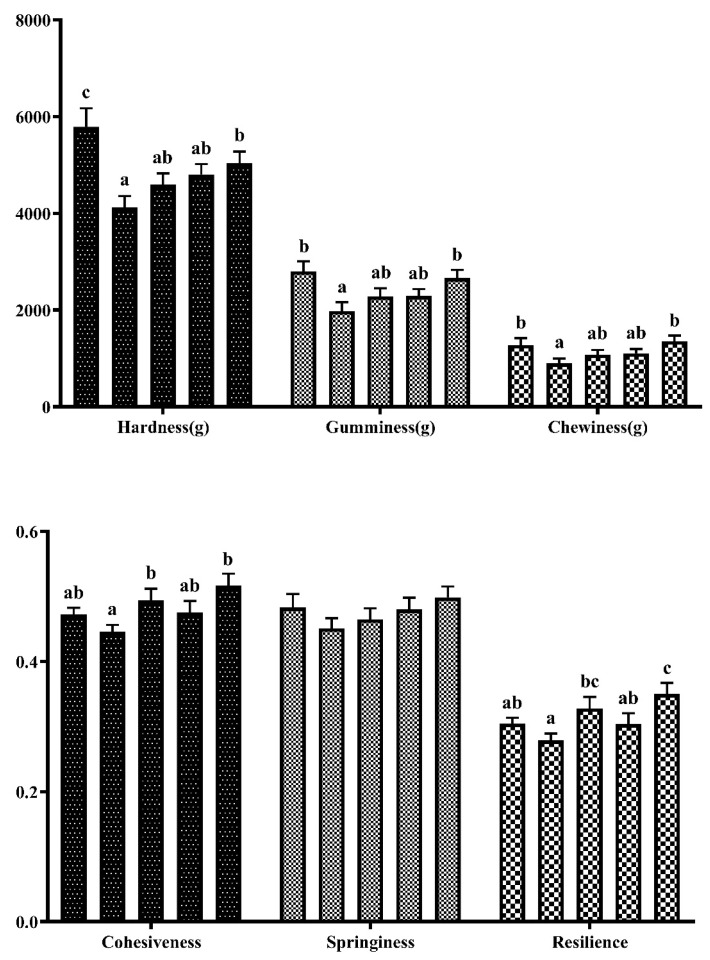
Back muscle texture of largemouth bass. ^abc^ Different groups with significant differences are represented by different letters, different groups without significant differences are represented by the same letter, and no letter means that there is no significant difference between all groups.

Figure 2 presents the results of the muscle texture analysis. Replacing 8.89% fishmeal in the diet (EHPB1) significantly reduced the hardness, gumminess, and chewiness of the largemouth bass back muscles (*p* < 0.05). Subsequently, the muscle hardness, gumminess, and chewiness increased as content of EHPB increased. Cohesiveness and resilience decreased in the EHPB1 group but showed an increasing trend as the EHPB content increased.

**Figure 3:**
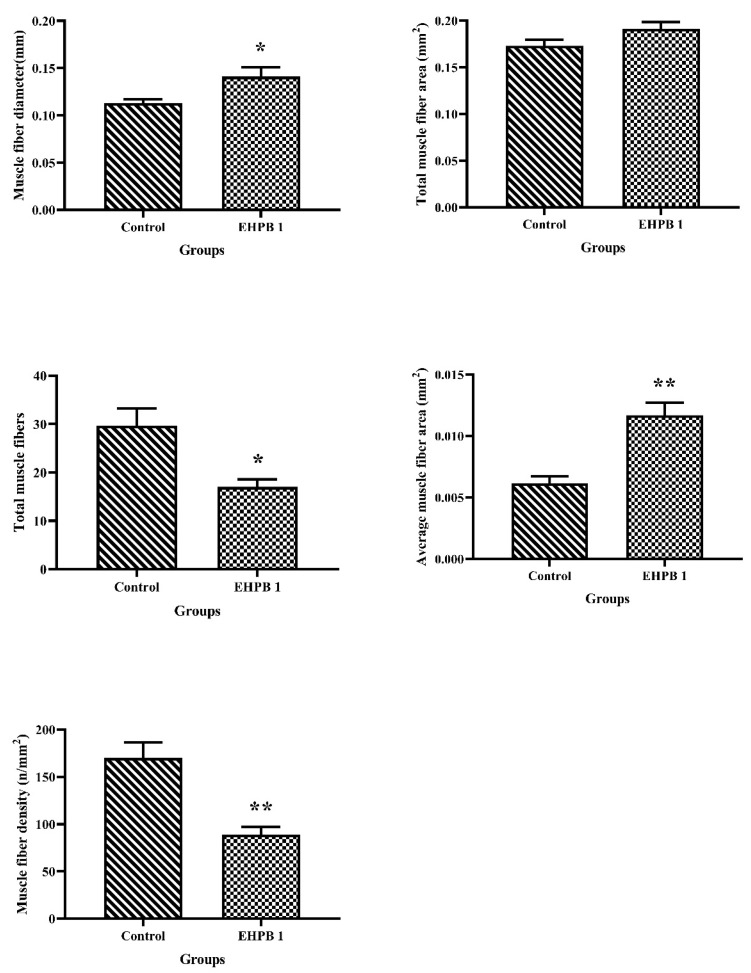
Measurement and analysis of back muscle cells of largemouth bass. Asterisk indicates the significant difference *p* value between groups, * indicates *p* < 0.05, ** indicates *p* < 0.01.

**Figure 4:**
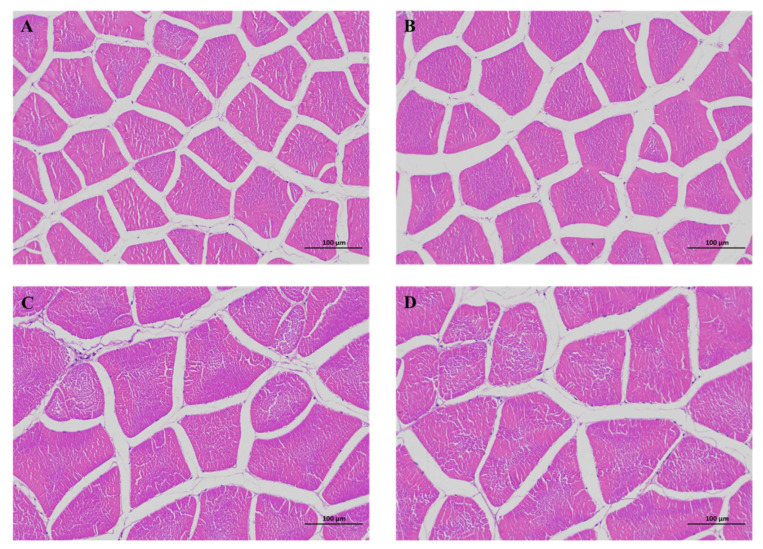
Back muscle slices of largemouth bass fed control (**A**,**B**) and EHPB 1 (**C**,**D**) diets were stained with HE (magnification 200).

Figure 3 and Figure 4 depict the results of largemouth bass back muscle slicing. The EHPB1 group showed significant increases in the diameter and average area of the back muscle fibers (*p* < 0.05), with significant decreases in the total number and density of muscle fibers (*p* < 0.05). Furthermore, the EHPB1 group exhibited a slight increase in the total back muscle area.

Table 6 shows the muscle composition of the control and EHPB1 groups. There were no significant differences in the crude composition between the two groups.

Table 7 presents the muscle findings of the analysis of amino acids and fatty acids in largemouth bass. The results showed that replacing 8.89% fishmeal with EHPB did not significantly affect the levels of various amino acids and fatty acids in largemouth bass.

### 3.4. Fluorescent Quantitative PCR

**Figure 5:**
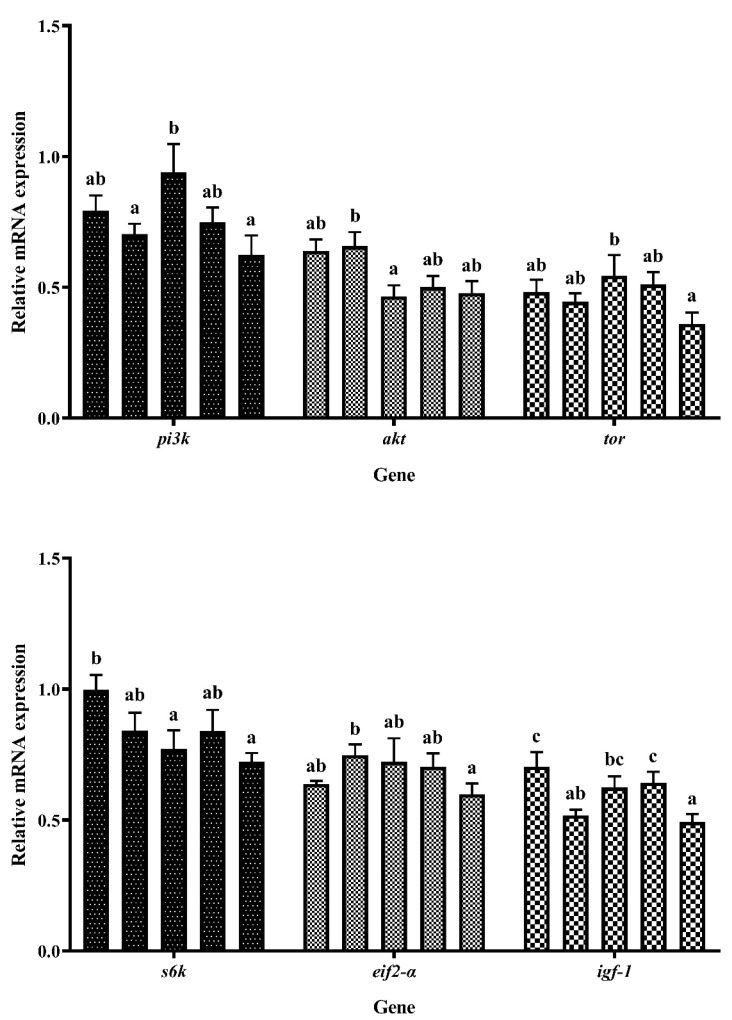
Expression of genes involved in protein synthesis in largemouth bass. ^abc^ Different groups with significant differences are represented by different letters, different groups without significant differences are represented by the same letter, and no letter means that there is no significant difference between all groups.

Figure 5 shows the expression of the protein synthesis-related genes. The dietary increase in EHPB content resulted in fluctuations in the expression of phosphatidylinositol 3-kinase (*pi3k*) and target of rapamycin (*tor*), with an initial increase followed by a decrease. The highest expression level was observed in the EHPB2 diet. The mRNA levels of protein kinase B (*akt*) were the highest in the EHPB1 group and lowest in the EHPB2 supplemented group. The increase in EHPB content resulted in a decrease in the mRNA levels of ribosomal protein S6 kinase polypeptide (*s6k*), and this decrease was significantly lower than that of the control group when the fish were fed the EHPB2 diet (*p* < 0.05). The expression of eukaryotic initiation factor-2α (*eif2-α*) initially increased and subsequently decreased in response to increasing EHPB content. The highest expression was observed in largemouth bass fed an EHPB1 diet. Compared with the control group, the expression of insulin-like growth factor-1(*igf-1*) decreased significantly in the EHPB1 group (*p* < 0.05), followed by an initial increase and then a decrease with increasing EHPB content.

**Figure 6:**
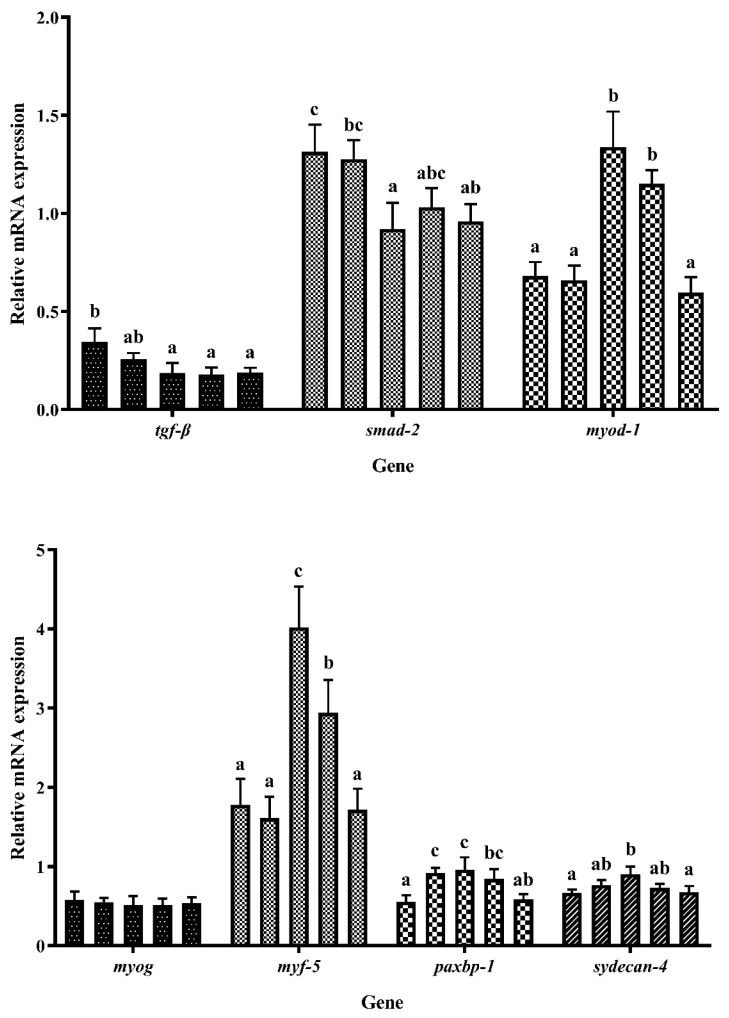
Expression of genes involved in muscle production. ^abc^ Different groups with significant differences are represented by different letters, different groups without significant differences are represented by the same letter, and no letter means that there is no significant difference between all groups.

Figure 6 illustrates the expression patterns of muscle production-related genes. The expression of transforming growth factor-β (*tgf-β*) decreased with increasing EHPB supplementation. At a fishmeal replacement level of 17.78% (EHPB2), the expression of *tgf-β* was significantly lower than that in the control group (*p* < 0.05). The increase in EHPB content resulted in fluctuations in the mRNA levels of smad-2 (*smad-2*), which initially decreased and subsequently increased. The lowest level was observed in EHPB2, which was significantly decreased compared with that in the control group (*p* < 0.05). In contrast, the mRNA levels of myogenic differentiation 1 (*myod-1*), myogenic factor 5 (*myf-5*), and syndecan-4 (*syndecan-4*) initially increased and then decreased, reaching the highest level when largemouth bass were fed the EHPB2 diet (*p* < 0.05). The mRNA levels of pax 3 and pax 7 binding protein 1 (*paxbp-1*) exhibited a similar trend but were significantly higher in the EHPB1 than in the control group. No significant differences were observed in myogenin (*myog*) expression.

**Figure 7:**
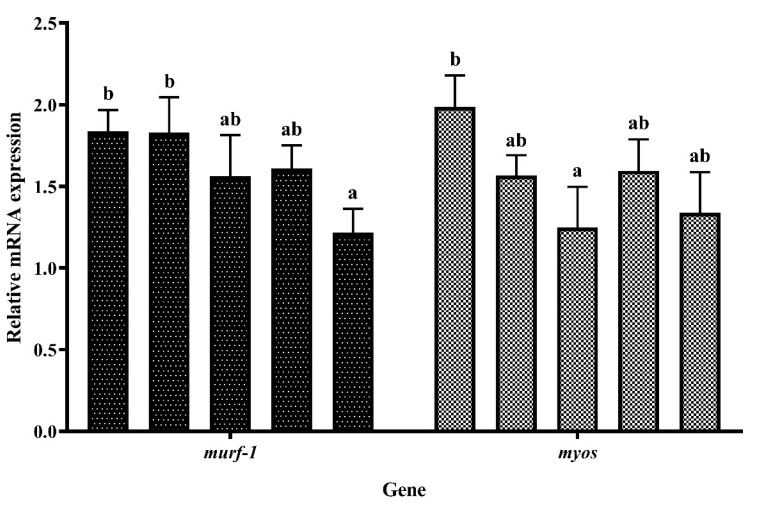
The expression of genes related to muscle atrophy in largemouth bass. ^ab^ Different groups with significant differences are represented by different letters, different groups without significant differences are represented by the same letter, and no letter means that there is no significant difference between all groups.

Figure 7 shows the expression of the muscle atrophy-related genes. Increasing dietary EHPB supplementation decreased the mRNA level of muscle ring-finger protein-1 (*murf-1*). The lowest level of *murf-1* expression was observed in the EHPB4 group, which was significantly lower than that in the control group (*p* < 0.05). Myostatin (*myos*) mRNA levels exhibited a biphasic pattern that initially decreased and subsequently increased. The lowest level was observed in EHPB2, which was significantly lower than that in the control group (*p* < 0.05).

## 4. Discussion

The replacement of fishmeal in aquaculture feed is a widely discussed topic for industrial advancement. Significant progress has been made in research on fishmeal replacements for aquatic feed [6,7,8]. This study revealed that replacing 8.89% fishmeal with EHPB promoted the growth performance of largemouth bass to a certain extent (refer to Table 4). However, as more fishmeal was replaced by EHPB, the growth performance of largemouth bass showed a significant decline. Similar to the results of this study, the partial replacement of fish meal in the Gibel carp (*Carassius auratus gibelio*) diet with certain poultry by-products promoted growth performance to a certain extent, but growth performance decreased with the increase in substitution [40]. Similarly, a small amount of replacement of fishmeal in the Florida pompano (*Trachinotus carolinus*) diet with poultry by-products did not negatively effect growth performance [41]. This confirms, to a certain extent, that higher levels of fishmeal substitution can lead to a decline in fish growth performance. Interestingly, in juvenile red drums (*Sciaenops ocellatus*), poultry byproducts negatively affect their body growth [42]. The observed discrepancy may be attributed to variations in the quantity of fishmeal replaced with poultry by-products in the formula and the specific type and size of the fish used in the study. Another possible explanation could be that enzymatic hydrolysis improves the absorption and utilization of poultry by-products. In conclusion, substituting 8.89% fishmeal with EHPB as a protein source could promote the growth of largemouth bass and reduce the feed coefficient rate. Plasma biochemistry serves as a crucial indicator for assessing the health status of fish, with any signs of fish being in an unhealthy condition reflected in their plasma [43]. In the plasma levels of this study, EHPB1 significantly increased ALB, ALT, AST, TC, GLU, and TP (refer to Table 5). Similar results were observed in largemouth bass fed protein derived from *Clostridium autoethanogenum* instead of fishmeal [44]. In contrast, levels of AST and TG in Pengze crucian carp (*Carassius auratus*) remained relatively stable when fed hydrolyzed feather meal instead of fishmeal, but the GLU concentration decreased [45]. Interestingly, the plasma biochemistry of olive flounder (*Paralichthys olivaceus*) is unaffected by the partial replacement of fishmeal with silkworm pupae meal, promate meal, and meat and bone meal [46]. One possible explanation is that the largemouth bass, which is naturally diabetic, experiences hyperglycemia and hyperproteinemia when fed a high-protein diet, resulting in increased levels of plasma TP and GLU, which subsequently causes an increase in other plasma markers [47,48]. One of the possible reasons for the change in plasma biochemical indexes was that EHPB contains more small molecules, which are more conducive to the digestion and absorption of fish, and thus was reflected in plasma [14]. Additionally, elevated levels of plasma biochemical indices such as GLU and TP suggest a higher metabolic rate in fish. The increased metabolism of EHPB1 may contribute to the successful substitution of fishmeal. Further research is needed to investigate the effect of EHPB on fish, particularly in relation to protein, glucose, and lipid metabolism, as the observed alterations in plasma biochemistry suggest potential effects in these areas.

Protein is a crucial component of aquatic feed and significantly contributes to its overall cost. In the current research, EHPB supplementation had a significant effect on the muscle quality of largemouth bass. Muscle hardness is strongly correlated with muscle texture [49,50]. It can directly affect various texture parameters and intuitively reflect muscle quality. In the structural analysis of largemouth bass back muscle, replacing only 8.89% fishmeal with EHPB in the diet significantly decreased the hardness, gumminess, and chewiness of the muscle, after which there was a trend of recovery (refer to Figure 2). In contrast, the texture parameters of Pengze crucian carp increased when they were fed hydrolyzed feather meal instead of fishmeal [45]. In addition, an increase in muscle texture was observed in grass carp (*Ctenopharyngodon idellus*) consuming paper mulberry, Atlantic salmon (*Salmo salar*) fed northern krill as a replacement for fishmeal, and grass carp consuming novel protein sources [51,52,53]. In this experiment, springing also shows a similar trend to the previous three. Although there is no significant effect, this similar trend seems to indicate that elasticity is closely related to hardness, gumminess, and chewiness. In our study, EHPB decreased cohesiveness and resilience. The lower texture was characterized by the presence of coarser muscle fibers and a reduced fiber count in certain sections (refer to Figure 3 and Figure 4). Similar results have been observed in tilapia fed broad beans [54]. Thinner muscle fibers in this section exhibited higher textural properties. However, other studies have reported conflicting results. A simultaneous increase in muscle stiffness and muscle fiber was found in red sea bream (*Pagrus major*) fed olive leaf powder and European sea bass (*Dicentrarchus labrax*) fed defatted yellow mealworm (*Tenebrio molitor*) larvae meal [55,56]. The findings of the present study indicate that largemouth bass supplemented with EHPB1 have larger fiber intervals in their dorsal muscle fibers (refer to Figure 3 and Figure 4). This could explain the decrease in texture parameters such as muscle hardness and mastication, among other factors. Furthermore, reduced muscle fiber density may also be a potential contributing factor to this result [57]. Moreover, replacing 8.89% fishmeal with EHPB did not affect the amino acid and fatty acid composition of largemouth bass muscle (Refer to Table 7). No significant effect was found on largemouth bass muscle composition by replacing 8.89% fishmeal with EHPB (refer to Table 6). No adverse effects were observed in this study when 8.89% fishmeal was replaced in the diet. Previous research has shown that replacing fish meal with poultry by-products can alter the amino acid and fatty acid composition of aquatic animals [58,59,60]. Enzymatic hydrolysis potentially increases the levels of free amino acids and small peptides, improving the utilization rate of raw materials in largemouth bass and mitigating the negative effects [61]. In summary, the analysis of muscle tissue and sections revealed that replacing 8.89% fishmeal with EHPB could significantly improve the muscle quality of largemouth bass without causing a loss of amino acids and fatty acids.

Physiological changes in aquatic animals frequently correlate with genetic alterations. Dietary EHPB had significant effects on both muscle composition and texture, as well as gene expression in largemouth bass. The analysis of mRNA related to protein metabolism-related pathways revealed that the replacement of 8.89% fishmeal with EHPB in the feed did not significantly affect protein metabolism-related genes (refer to Figure 5). This coincides with the results of muscle protein content (refer to Table 7). However, only *igf-1* expression was found to be significantly reduced. *igf-1* regulates protein metabolism and muscle cell production [62]. The results in this experiment suggest that downregulation of *igf-1* expression may contribute to the reduction in largemouth bass muscle cell count. Variations in the quantity of largemouth bass muscle fibers further affect their muscle tissue texture. To further explore the factors influencing muscle cell growth, we identified gene expression patterns associated with muscle production. *tgf-β* and *smad-2* have inhibitory effects on muscle cell growth and stimulatory effects on muscle cell proliferation [63,64]. In this study (refer to Figure 6), we observed a slight decrease in the expression levels of *tgf-β* and *smad-2* in the EHPB1 group, which was statistically significant in the EHPB2 group. This could explain the reduction in muscle cell count and increase in the cross-sectional area in the EHPB1 group. The reduction in the muscle fiber count may have contributed to the alteration of the texture parameters in this study. Myogenic regulatory factors, including *myod-1*, *myf-5,* and *myog*, are involved in the regulation of muscle production [65,66]. In the current study, no significant effect on *myog* was observed when the amount of EHPB replacing fishmeal in feed was increased. The mRNA levels of *myod-1* and *myf-5* were significantly elevated in the EHPB2 and EHPB3 groups compared to those in the control group. This suggests that a higher content of EHPB should be included in the diet than EHPB1 to influence alterations in texture parameters across these three factors. In this study, EHPB1 supplementation significantly increased *paxbp-1* mRNA content and *syndecan-4* expression in the largemouth bass muscle (refer to Figure 7). *paxbp-1* regulates the cell growth checkpoint that controls muscle cell hypertrophy [67]. Simultaneously, *syndecan-4* overexpression induces muscle cell hypertrophy [68]. This finding explains the reason for muscle cell enlargement in the muscle section samples of largemouth bass in the EHPB1 group. In addition, the expression levels of *murf-1* and *myos* in the muscle of largemouth bass fed an EHPB1 diet were not significantly affected. *murf-1* mediates muscle cell protein degradation and regulates muscle cell reduction [69]. *myos* is a potent inducer of muscle atrophy and can inhibit muscle development [70]. This finding aligns with the results of our muscle-slice experiment.

## 5. Conclusions

In summary, 3.10% EHPB can be used to replace 8.89% fishmeal in the feed without any adverse effects on growth. Replacing 8.89% of fishmeal with EHPB improved largemouth bass muscle quality (texture and nutrition of muscle) without any adverse effects on the composition of muscle amino and fatty acids. This study demonstrated the effect of fishmeal replacement by EHPB on largemouth bass muscle quality, provided guidance for reducing the use of fishmeal in aquatic feed and improving the muscle quality of aquatic products, and provided some help for the subsequent production practice.

## Figures and Tables

**Table 1:** Formulation (% dry matter).

Diet	Control	EHPB 1	EHPB 2	EHPB 3	EHPB 4
Replace fishmeal levels	0.00	8.89	17.78	26.67	35.56
Enzymatic hydrolysis of poultry by-product ^a^	0.00	3.10	6.20	9.30	12.40
Fish meal ^a^	45.00	41.00	37.00	33.00	29.00
Blood meal ^a^	6.00	6.00	6.00	6.00	6.00
Soybean meal ^a^	13.00	13.00	13.00	13.00	13.00
Corn gluten meal ^a^	3.00	3.00	3.00	3.00	3.00
Wheat flour ^a^	7.00	7.00	7.00	7.00	7.00
Tapioca starch	7.00	7.00	7.00	7.00	7.00
Rice bran	7.52	7.52	7.52	7.52	7.52
Microcrystalline cellulose	2.23	2.31	2.43	2.61	2.69
Squid paste	2.00	2.00	2.00	2.00	2.00
Fish oil	3.70	3.99	4.28	4.47	4.76
Vitamin premix ^b^	1.00	1.00	1.00	1.00	1.00
Mineral premix ^b^	1.00	1.00	1.00	1.00	1.00
Calcium dihydrogen phosphate	1.00	1.45	1.85	2.30	2.75
Vitamin C	0.05	0.05	0.05	0.05	0.05
Choline chloride	0.50	0.50	0.50	0.50	0.50
L-Lysine ^c^	0.00	0.05	0.10	0.16	0.21
L-Methionine ^c^	0.00	0.03	0.06	0.09	0.12
Total	100.00	100.00	100.00	100.00	100.00
Taurine (mg/kg)	0.00	11.40	22.80	34.20	45.60
Proximate analysis (% of dry diet)					
Crude protein (%)	49.81	50.06	49.25	49.11	50.60
Crude lipid (%)	11.2	11.2	11.2	11.1	11.1

^a^ All ingredients obtained from Wuxi Tongwei feedstuffs Co., Ltd. (Wuxi, China). ^b^ Premixes were obtained from HANOVE Animal Health Products Co., Ltd. (Wuxi, China). ^c^ Dietary supplementation of limiting amino acids (L-lysine and L-methionine) to compensate for deficiencies in EHPB.

**Table 2:** Test related instruments and reagents.

Instrument	Type	Manufacturer
Real-time PCR machine	7500 real-time PCR machine	Applied Biosystems, Carlsbad, NM, USA
Microspectrophotometer	Nano Drop 2000	Thermo Fisher Multiskan GO, Shanghai, China
Automatic analyzer	BS-400	Mindray, Shenzhen, China
Auto Kjeldahl apparatus	Hanon K1100	Jinan Hanon Instruments Co., Ltd., Jinan, China
Muffle	XL-2A	Hangzhou Zhuochi Instrument Co., Ltd., Hangzhou, China
Photographic microscope	Eclipse Ci-L	Nikon, Tokyo, Japan
Amino acid analyzer	SYKAM S-433D	Sykam GmbH, Munich, Germany
**Reagent**		
RNA isolator	R401-01-AA	Vazyme, Nanjing, China
HiScript^®^ II One Step qRT-PCR SYBR Green	Q221-01	Vazyme, Nanjing, China
ALB	BS-400	Mindray Medical International Ltd., Shenzhen, China (Include kits and machine)
ALT		
AST		
TC		
TG		
GLU		
TP		
ALP		

Plasma alkaline phosphatase (ALP), albumin (ALB), alanine aminotransferase (ALT), aspartate aminotransferase (AST), plasma glucose (GLU), triglyceride (TG), total cholesterol (TC), total protein (TP).

**Table 3:** Primer sequence.

Gene	Forward Sequence	Reverse Sequence	Accession Number/ Reference
*pi3k*	CTCACCATGGAGGATGGACC	ACGGTGGGAGTGGAGGTTTA	Cluster-21914.23096
*akt*	AGCGCACCTTCCATGTAGAC	GGCTATTTGCCACTTGCTGG	AXE72881.1
*tor*	CCATCCTCAACCTACTTCC	CTCTCCTTCTCCTTCTTCAG	Cluster-21914.16479
*s6k*	GTAATGCAAAGGACACGGCG	GTTCCCCACCGCTCAGATAC	XP_010747297.3
*eif2-α*	CCTCGTTTGTCCGTCTGTATC	GCTGACTCTGTCGGCCTTG	XM_038728156.1
*igf-1*	CCTCTGCCTGTGTATAATCA	TGTCCGTCTTAGCCATCT	XM_038738328.1
*tgf-β*	GCTCAAAGAGAGCGAGGATG	TCCTCTACCATTCGCAATCC	[37]
*smad-2*	ATTCTGACAGAGCTTCCGCC	ATTTCTGCTGGTGAGCCTGTT	XM_038733539.1
*myod-1*	CCTGCTGTTACTGCTCTG	ACCACTGATGTCCACTGA	XM_038706745.1
*myog*	TGACTTGTAACTCTGCTGAT	ATGTCTGGATGGTAGGATAAG	XM_038697403.1
*myf-5*	CAACTTTGTGGACCGCAGAC	CCTGCTCTCGTAACAGGTCC	XM_038738312.1
*paxbp-1*	GCCTCAGTTGGAGCCATTCT	TGATGTGGTCCAGGGCATTC	XM_038708491.1
*snydecan-4*	TCCAAGACATCCGCTAAGCC	GATCTCCACCTCGTTGACGG	XM_038697979.1
*murf-1*	AGAACACGGACCTACAGAG	CGGCTTGGTGAACATCTC	XP_018534138.1
*myos*	ACCTTGGAGTGAATGTAGAC	GAGTGGAGTGGAGTGGAT	DQ666527.3
*β-actin*	ATGCAGAAGGAGATCACAGCCT	AGTATTTACGCTCAGGTGGGG	MH018565.1

*pi3k*, phosphatidylinositol 3-kinase; *akt*, protein kinase B; *tor*, target of rapamycin; *s6k*, ribosomal protein S6 kinase-polypeptide; *eif2-α*, eukaryotic initiation factor-2α; *igf-1*, insulin-like growth factor-1; *tgf-β*, transforming growth factor-β; *smad-2*, smad-2; *myod-1*, myogenic differentiation 1; *myog*, myogenin; *myf-5*, myogenic factor 5; *paxbp-1*, pax 3 and pax 7 binding protein 1; *syndecan-4*, syndecan-4; *murf-1*, muscle ring-finger protein-1; *myos*, myostatin; *β-actin*, beta-actin.

**Table 4:** Growth performance and feed utilization of largemouth bass.

Diet	Control	EHPB 1	EHPB 2	EHPB 3	EHPB 4
IBW (g)	240.33 ± 0.17	240.17 ± 0.17	240.00 ± 0.00	240.17 ± 0.17	240.17 ± 0.17
FBW (g)	437.03 ± 11.35 ^c^	440.00 ± 1.53 ^c^	418.13 ± 1.62 ^b^	406.40 ± 3.85 ^ab^	397.20 ± 0.85 ^a^
SGR (%/day)	0.88 ± 0.04 ^c^	0.89 ± 0.00 ^c^	0.82 ± 0.01 ^b^	0.77 ± 0.01 ^ab^	0.74 ± 0.00 ^a^
WGR (%)	81.85 ± 4.85 ^c^	83.21 ± 0.56 ^c^	74.22 ± 0.67 ^b^	69.21 ± 1.55 ^ab^	65.39 ± 0.42 ^a^
FCR	1.21 ± 0.05 ^a^	1.24 ± 0.00 ^ab^	1.26 ± 0.03 ^ab^	1.34 ± 0.04 ^b^	1.47 ± 0.01 ^c^
SR (%)	96.67 ± 3.33 ^ab^	100.00 ± 0.00 ^b^	96.67 ± 3.33 ^ab^	93.33 ± 3.33 ^ab^	90.00 ± 0.00 ^a^

IBW: initial body weight = sum of initial body total weight per tank/number of fish in tank at beginning of experiment. FBW: final body weight = sum of final body total weight per tank/number of fish in tank at end of experiment. SGR: specific growth rate = 100 × ((ln (final body average weight (g))—ln (initial body average weight (g)))/days). WGR: weight gain = 100 × (final body average weight (g)—initial body average weight (g))/initial weight (g). FCR: feed conversion ratio = dry feed fed (g)/wet weight gain (g). SR: survival rate = 100 × (final number of fish/initial number of fish). ^abc^ Different groups with significant differences are represented by different letters, different groups without significant differences are represented by the same letter, and no letter means that there is no significant difference between all groups.

**Table 5:** Plasma biochemical analysis of largemouth bass.

	Control	EHPB 1	EHPB 2	EHPB 3	EHPB 4
ALB (g/L)	12.33 ± 1.25 ^a^	16.35 ± 1.21 ^b^	15.48 ± 0.56 ^ab^	24.07 ± 1.85 ^c^	13.75 ± 0.59 ^ab^
ALT (U/L)	2.00 ± 0.38 ^a^	3.76 ± 0.70 ^b^	2.22 ± 0.78 ^a^	1.65 ± 0.18 ^a^	1.24 ± 0.47 ^a^
AST (U/L)	9.74 ± 2.16 ^a^	22.65 ± 2.31 ^b^	16.61 ± 4.74 ^ab^	16.51 ± 2.41 ^ab^	18.36 ± 2.63 ^ab^
TC (mmol/L)	8.06 ± 0.33 ^a^	10.22 ± 0.50 ^b^	9.01 ± 0.47 ^ab^	8.85 ± 0.42 ^ab^	9.37 ± 0.53 ^ab^
TG (mmol/L)	6.58 ± 0.60 ^ab^	7.80 ± 0.26 ^b^	6.88 ± 0.50 ^ab^	6.89 ± 0.45 ^ab^	5.66 ± 0.55 ^a^
GLU (mmol/L)	7.47 ± 0.44 ^a^	10.06 ± 0.71 ^b^	6.44 ± 0.59 ^a^	7.47 ± 0.73 ^a^	7.99 ± 0.63 ^a^
TP (g/L)	42.73 ± 2.92 ^a^	49.36 ± 0.87 ^b^	51.97 ± 2.05 ^b^	50.25 ± 1.85 ^b^	46.22 ± 2.13 ^ab^
ALP (U/L)	69.94 ± 4.20 ^c^	63.54 ± 4.40 ^bc^	49.91 ± 4.80 ^a^	53.16 ± 3.72 ^ab^	63.89 ± 3.28 ^bc^

Albumin (ALB), alanine aminotransferase (ALT), aspartate aminotransferase (AST), total cholesterol (TC), triglyceride (TG), glucose (GLU), total protein (TP), and alkaline phosphatase (ALP). ^abc^ Different groups with significant differences are represented by different letters, different groups without significant differences are represented by the same letter, and no letter means that there is no significant difference between all groups.

**Table 6:** Muscle composition of control and EHPB1 groups.

Group	Item			
	Moisture (%)	Crude Protein (% W.W.) ^1^	Crude Lipid (% W.W.) ^1^	Crude Ash (% W.W.) ^1^
Control	77.22 ± 0.21	18.19 ± 0.01	3.57 ± 0.10	1.22 ± 0.05
EHPB1	77.55 ± 0.24	18.02 ± 0.05	3.81 ± 0.07	1.24 ± 0.02

^1^ W.W.: wet weight.

**Table 7:** Muscle amino acid and fatty acid contents of largemouth bass.

Amino Acid (%)	Control	EHPB 1	Fatty Acid (%)	Control	EHPB 1
Methionine	2.687	2.675	C14:0	1.56 ± 0.17	1.66 ± 0.17
Cystine	0.766	0.772	C15:0	0.21 ± 0.01	0.20 ± 0.00
Methionine + cystine	3.463	3.447	C16:0	17.35 ± 0.65	18.95 ± 0.45
Lysine	8.281	8.136	C16:1	4.33 ± 0.07	4.49 ± 0.32
Threonine	3.936	3.935	C17:0	0.22 ± 0.02	0.27 ± 0.03
Tryptophan	/	/	C18:0	4.15 ± 0.89	4.43 ± 0.31
Arginine	5.224	5.212	C18:1	24.70 ± 1.50	28.45 ± 2.95
Isoleucine	4.415	4.391	C18:2	24.15 ± 4.45	21.90 ± 0.60
Leucine	7.293	7.263	C18:3n6γ	/	/
Valine	4.604	4.604	C18:3n3α	1.87 ± 0.33	1.73 ± 0.06
Histidine	2.213	2.123	C20:0	/	/
Phenylalanine	4.12	4.192	C20:1	0.82 ± 0.01	1.15 ± 0.42
Glycine	4.559	4.384	C20:2	0.75 ± 0.02	0.82 ± 0.11
Serine	3.445	3.424	C20:3n6	0.47 ± 0.00	0.31 ± 0.08
Proline	3.162	3.151	C20:4n6	1.49 ± 0.40	1.06 ± 0.40
Alanine	5.496	5.427	C20:5n3	1.72 ± 0.32	1.35 ± 0.20
Aspartic acid	9.292	9.285	C23:0	/	/
Glutamic acid	13.05	13.014	C24:1	/	/
Ammonia	1.288	1.339	C22:6n3	16.30 ± 4.20	13.15 ± 2.65
Total amino acids (including ammonia)	83.841	83.327	Total fatty acid	99.99 ± 0.01	99.93 ± 0.02
Total amino acids (excluding ammonia)	82.553	81.988			

## Data Availability

The authors confirm that the data supporting the findings of this study are available within the manuscript, figures, and tables.
